# Non-compliance with smoke-free law in public places: a systematic review and meta-analysis of global studies

**DOI:** 10.3389/fpubh.2024.1354980

**Published:** 2024-04-17

**Authors:** Chala Daba, Amanuel Atamo, Kassahun Ayele Gasheya, Abebe Kassa Geto, Mesfin Gebrehiwot

**Affiliations:** ^1^Department of Environmental Health, College of Medicine and Health Sciences, Wollo University, Dessie, Ethiopia; ^2^Department of Occupational Health and Safety, College of Medicine and Health Sciences, Wollo University, Dessie, Ethiopia; ^3^Department of Nursing and Midwifery, Dessie Health Science College, Dessie, Ethiopia

**Keywords:** active smokers, non-compliance, public place, smoke-free law, secondhand smoke

## Abstract

**Introduction:**

Non-compliance with smoke-free law is one of the determinants of untimely mortality and morbidity globally. Various studies have been conducted on non-compliance with smoke-free law in public places in different parts of the world; however, the findings are inconclusive and significantly dispersed. Moreover, there is a lack of internationally representative data, which hinders the evaluation of ongoing international activities towards smoke-free law. Therefore, this meta-analysis aimed to assess the pooled prevalence of non-compliance with smoke-free law in public places.

**Methods:**

International electronic databases, such as PubMed/MEDLINE, Science Direct, Cochrane Library, CINAHL, African Journals Online, HINARI, Semantic Scholar, google and Google Scholar were used to retrieve the relevant articles. The study followed the Preferred Reporting Items for Systematic Reviews and Meta-Analysis Protocols (PRISMA) guidelines. The Higgs I^2^ statistics were used to determine the heterogeneity of the reviewed articles. The random-effects model with a 95% confidence interval was carried out to estimate the pooled prevalence of non-compliance.

**Results:**

A total of 23 articles with 25,573,329 study participants were included in this meta-analysis. The overall pooled prevalence of non-compliance with smoke-free law was 48.02% (95% CI: 33.87–62.17). Extreme heterogeneity was observed among the included studies (I^2^ = 100%; *p* < 0.000). The highest non-compliance with smoke-free law was noted in hotels (59.4%; 95% CI: 10.5–108.3) followed by homes (56.8%; 95% CI: 33.2–80.4), with statistically significant heterogeneity.

**Conclusion:**

As the prevalence of non-compliance with smoke-free law is high in public places, it calls for urgent intervention. High non-compliance was found in food and drinking establishments and healthcare facilities. In light of these findings, follow-up of tobacco-free legislation and creating awareness that focused on active smokers particularly in food and drinking establishments is recommended.

## Introduction

Exposure to secondhand smoke is a significant cause of premature morbidity and mortality worldwide. According to the World Health Organization report, more than 8 million people die annually as a result of tobacco-related diseases. Of these, 1.3 million deaths were due to exposure to secondhand smoke (1). Specifically, in the United States of America, secondhand smoke is responsible for the death of 41,000 people each year (2). Besides, mortality and morbidity, secondhand smoke has also a significant effect on economic development. For instance, evidence from recent reports showed that more than $1.4 trillion is lost due to the treatment of tobacco-related problems (3, 4).

The health effects of exposure to secondhand smoke are more prevalent in developing countries. More than 80% of the global tobacco-related deaths were in low and middle-income countries (1). Global adult tobacco survey in Pakistan showed 16.8 million (70%) and 21.2 million (90%) adults were exposed to secondhand smoke at workplaces and restaurants, respectively (5). Similarly, 1.2 million Bangladesh people were affected by tobacco-related diseases (6). As a result, more than 61,000 children experienced diseases in 2018 alone (7). Evidence from a recent study also revealed that the health risks associated with exposure to secondhand smoke are high in South and Southeast Asia (8). Similarly in Ethiopia, 27.1 and 7.0% of the population are exposed to secondhand smoke at their workplaces and healthcare facilities, respectively (9). As Ezzati et al. (10), suggest, exposure to secondhand smoke could lead to a high proportion of lung cancer (71%), chronic respiratory diseases (42%), and cardiovascular disease (10%).

To reduce the harmful effects of secondhand smoke exposure, WHO recommends the development of 100% smoke-free law in public places (11). In order to prevent secondhand smoke exposure in public places, parties must take increased and ongoing measures, according to Article 8 of the WHO framework convention on tobacco control (12). Notwithstanding the compressive interventions that have been taken to create smoke-free environment across the world, the prevalence of cigarette smoking and exposure to secondhand smoke is still increasing in different countries except Brazil and Turkey (13). For instance, evidence from recent studies showed that the prevalence of non-compliance with smoke-free law was as high as 87.9, 79, 67.1, and 41.2% in public places of Ethiopia (14), United States (15), Indonesia (16), and China (17), respectively, 39.9% among patients in Spain (18), 39.1% in healthcare facilities of Australia (19), and 32% among healthcare workers in the United Kingdom (20).

Numerous studies have been carried out on non-compliance with smoke-free law across the world (15–17, 19, 21–38). However, the findings are inconclusive, which could hinder the evaluation of ongoing interventions and the redesign of other effective interventional activities. Moreover, there is no global study assessing the pooled prevalence of non-compliance with smoke-free law in public places. Therefore, this systematic review and meta-analysis aimed to estimate the pooled prevalence of non-compliance with smoke-free law in different public places. The findings from this meta-analysis would help to identify potential public place violations and guide policy enforcement measures to reduce the burden of tobacco-related mortality and morbidity.

## Materials and methods

### Study registration

The protocol for this systematic review has been registered in the International Prospective Registry of Systematic Reviews (PROSPERO) under the registration number CRD42023444710.

### Study selection, search strategy, and study period

Relevant studies were retrieved from electronic databases, such as PubMed/MEDLINE, Science Direct, Cochrane Library, CINAHL, African Journals Online, HINARI, and Sematic Scholar. Google and Google Scholar searches were also used to search for relevant articles. Besides, gray literatures were also identified from different university’s digital libraries and published articles. The following key terms were used to search the studies: “non-compliance,” “compliance,” “smoke-free law,” “smoking-ban,” “smoking ban,” “smoke-free legislation,” “associated factors,” “factors,” “determinant factors,” “factors associated,” “public institution,” “home,” “restaurant,” “cafe,” “hospitals,” “schools,” “bar,” “bar and restaurant” and “public place.” All key terms were combined using the Boolean operators “AND” or “OR” as appropriate (Supplementary material S1). The search was carried out up to November 23, 2023 by four authors independently (CD, MG, AKG, and AA). Those studies searched from selected databases were transferred to Endnote version 20 and duplicate files were excluded. The process of selecting these studies was following the Preferred Reporting Items for Systematic Reviews and Meta-Analysis (PRISMA) guidelines (39) (Supplementary material S2).

### Inclusion and exclusion criteria

*Population*: This meta-analysis includes global studies conducted on smoke-free law in public places.
*Exposure*: Public places that did not comply with smoke-free law.
*Comparison*: Public places that comply with smoke-free law.
*Outcome*: Studies assessed non-compliance with smoke-free law as the primary outcome.
*Study setting*: Institutional-based studies.
*Study design*: All observational studies (cohort, cross-sectional, and case–control).
*Publication*: Published studies were included.
*Country*: Studies conducted across the world.
*Language*: Studies published only in the English language were included in the review.
*Year of publication*: Studies published from 2000 to November 23, 2023.

### Exclusion criteria

Studies without full text, qualitative studies, irretrievable studies, letters-to-the editor, studies with poor methodological quality, and studies that did not report the outcome of interest were excluded from the meta-analysis.

### Outcome assessment

The primary outcome of the study was to estimate the pooled prevalence of non-compliance with smoke-free law in public places, determined by dividing the number of public places/smokers by the total sample size and multiplying by 100.

### Data extraction and risk of bias assessment

Three authors (CD, AA, and MG) independently extracted all the necessary data using a standard data extraction template. The data extraction template consisted of various study details, such as author’s name, country, publication year, type of public place, study design, methods of data collection, response rate, and prevalence. Three reviewers (CD, AA, and AKG) screened the relevant articles for inclusion after duplicate files were removed. The quality of each article was evaluated using the Joana Brigg Institute (JBI) critical appraisal checklist for cross-sectional studies (40) (Supplementary material S3). Two authors (CD and AA) independently assessed the quality of each article, with scores measured on a scale of 100%. A quality score of greater than 50% was used to include articles for further analysis (41, 42). In the case of any discrepancies encountered during the quality assessment, the mean score was computed from the evaluations of all reviewers.

### Statistical analysis

The level of heterogeneity among the included studies was statistically evaluated using the Higgs I^2^ test, with values of 25, 50, and 75% indicating low, moderate, and high heterogeneity, respectively (43). Because high heterogeneity was observed among the included studies (I^2^ = 100%, *p* < 0.000), DerSimonian and Liard (44) method of random-effects model was used to estimate the pooled prevalence of non-compliance with smoke-free law in public places.

Forest plot was used to present the pooled prevalence of non-compliance. A sensitivity analysis was performed to assess the influence of a single study on the pooled prevalence estimates. Subgroup analysis was also conducted based on various study characteristics, such as year of publication (before 2020 or 2020 and after), sample size (small- < 1000 or large- ≥1000) and methods of data collection (observation, self-administration or review document). Besides, publication bias was assessed using a funnel plot and Egger’s test with a *p*-value less than 0.05 suggesting a publication bias (45). Moreover, meta-regression analysis was also carried out considering variables, such as year of publication, sample size and method of data collection to the outcome variable.

## Results

### Study selection

**Figure 1:**
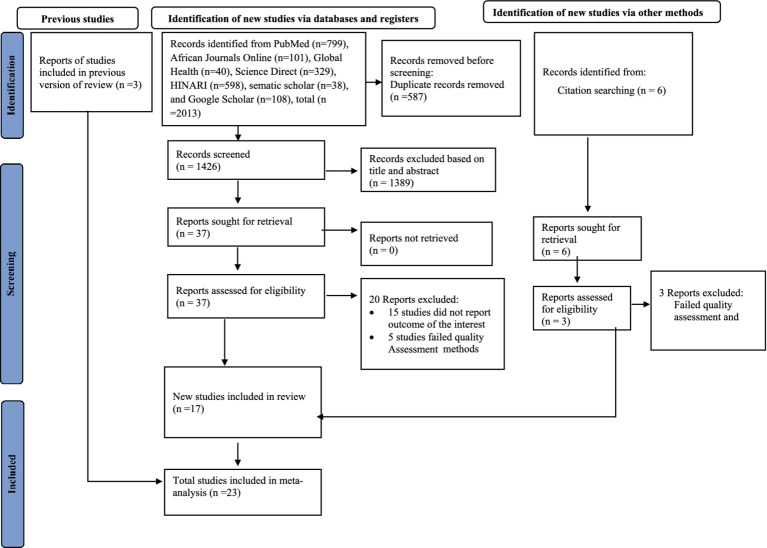
PRISMA flow diagram of the included studies for the systematic review and meta-analysis of smoke-free law non-compliance in public places, 2023.

A total of 2013 articles were identified from an international database. Using the Endnote reference manager, 587 duplicate articles were excluded; while 1,389 were excluded as they do not meet the inclusion criteria based on their titles and abstracts. Besides, 20 articles were excluded based on the quality of the assessment and the outcomes of the studies. Finally, 23 full-text articles were eligible for this meta-analysis and the processes of selecting these studies followed the Preferred Reporting Items for Systematic Reviews and Meta-Analysis (PRISMA) guideline (Figure 1).

### Characteristics of the included studies

In this meta-analysis, 25,573,329 study participants were involved. Five of the included studies focused on healthcare facilities (19, 34–37), sixteen studies (14–17, 21–27, 29–31, 33, 38), one study on education sector (38), and one study on food and drinking service establishment (32). All of the included studies followed cross-sectional study design. From the included studies, the highest non-compliance with smoke-free law was found to be 98.2% in food and drinking establishments (32) and the lowest non-compliance was 10.3% in healthcare facilities (36). Regarding the study country, four studies were conducted in India (29, 31, 35, 37), two in China (17, 30), two in Russia (28, 38), one in United states (15), one in Guatemala (24) one in Indonesia (16), two in Ethiopia (14, 36), two in South Africa (23, 32), one in Australia (19), one in Portugal (34), one in European Union (27), one in Bangladesh (26), one in Vietnam (33), one in Turkey (22), one in Pakistan (21), and one in Nepal (25). Among the included the studies, the sample size ranged between 25,475,032 (15) and 40 (37) (Table 1).

**Table 1:** Descriptive summary of twenty-three studies included to estimating the pooled prevalence of non-compliance with smoke-free law in public places, 2023.

Authors	Year of publication	Country	Methods of data collection	Study design	Study setting	Sample size	Prevalence (%)	Quality score (%)
McCrabb et al. (19)	2017	Australia	Self-reported online	Cross-sectional	Hospital	805	39.1	75
Hoe et al. (30)	2021	China	Observational	Cross-sectional	All public places	694	35.3	87.5
Rijhwani et al. (35)	2018	Delhi	Observational	Cross-sectional	Hospital	155	55	87.5
Tadesse et al. (36)	2019	Ethiopia	Observational	Cross-sectional	Hospital	354	10.3	100
Filippidis et al. (27)	2015	EU	Self-administration	Cross-sectional	All public places	26,751	29	62.5
Tripathy et al. (37)	2013	India	Observational	Cross-sectional	Healthcare	40	77	87.5
Kumar et al. (31)	2014	India	Review report	Cross-sectional	All public places	20,455	49	87.5
Basnet et al. (25)	2022	Nepal	Observational	Cross-sectional	All public places	725	43.6	100
Galimov et al. (28)	2018	Russia	Review report	Cross-sectional	Education	716	40	87.5
Zasimova et al. (38)	2019	Russia	Review report	Cross-sectional	All public places	4,006	27.2	87.5
Ayo-Yusuf et al. (23)	2014	South Africa	Review report	Cross-sectional	All public places	3,094	55.9	75
Ay et al. (22)	2016	Turkey	observational and interview	Cross-sectional	All public places	450	29.7	75
Reis et al. (34)	2014	Portugal	observational	Cross-sectional	Healthcare	1,412	24.1	87.5
Suarjana et al. (16)	2020	Indonesia	Observational	Cross-sectional	All public places	538	67.1	62.5
Mengesha et al. (14)	2023	Ethiopia	Observational	Cross-sectional	All public places	1,282	87.7	100
Donahoe et al. (15)	2018	United States	Review report	Cross-sectional	All public places	25,475,032	79	62.5
Barnoya et al. (24)	2016	Guatemala	Sampling	Cross-sectional	All public places	41	71	87.5
Goel et al. (29)	2017	India	Observational	Cross-sectional	All public places	7,400	16.2	87.5
Nemakhavhani et al. (32)	2016	South Africa	Observational	Cross-sectional	FDE	56	98.2	75
Nguyen et al. (33)	2019	Vietnam	Observational	Cross-sectional	All public places	8,996	13.23	75
Chowdhury et al. (26)	2023	Bangladesh	Observational	Cross-sectional	All public places	313	73.5	100
Yang et al. (17)	2016	China	Self-administration	Cross-sectional	All public places	18,310	41.2	75
Ahsan et al. (21)	2022	Pakistan	Observation	Cross-sectional	All public places	1,704	43	87.5

Hint: all public places means at least three public places (hospitals, bar, food and drinking establishments, home, workplace, and education).

Regarding methods of data collection, thirteen studies collected data using observational methods (14, 16, 21, 25, 26, 29, 30, 32–37), five studies using review report (15, 23, 28, 31, 38), three studies using self-administered questionnaire (17, 19, 27), one study using both observational and interview (22), and one study collected using data measuring (24) (Table 1).

### Meta-analysis

**Figure 2:**
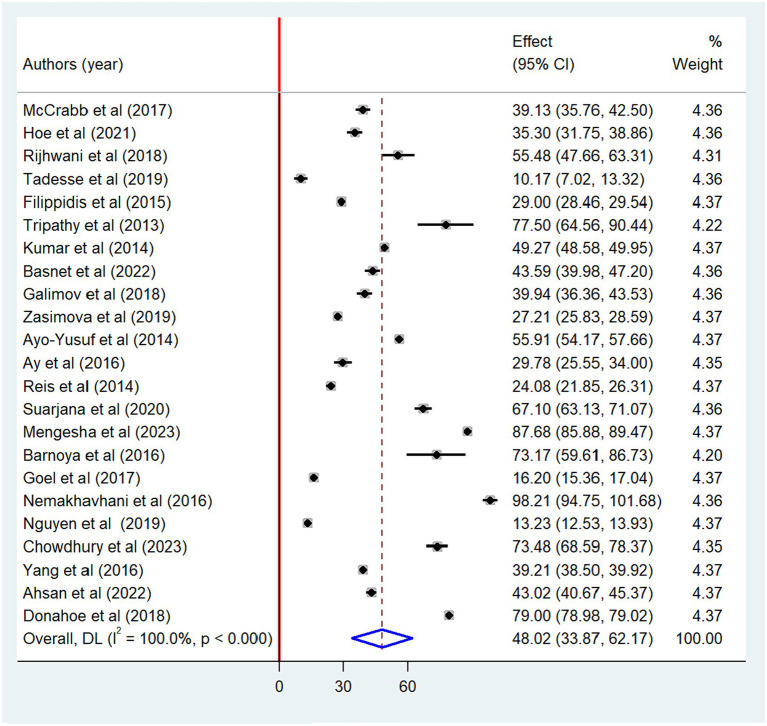
Forest plot of the pooled smoke-free law non-compliance in public places, 2023.

We found that the pooled prevalence of non-compliance with the smoke-free law is 48.02% (95% CI: 33.87–62.17) (Figure 2). Among the types of public places, the highest pooled prevalence of non-compliance was observed in hotels (59.4%) followed by homes (56.8%) (Table 2).

**Table 2:** Pooled non-compliance-free law among different public places, 2023.

Types of public places	Number of studies	Pooled non-compliance (95% CI)	Heterogeneity	
			I^2^	*p*-value
Healthcare facility	8	44.5 (31.4–57.3)	98.8%	<0.000
Education	6	35.2 (18.1–52.2)	99.6%	<0.000
Bar	5	49.1 (30.6–67.6)	100%	<0.000
Restaurant	7	32.6 (2.89–61.3)	100%	<0.000
Hotel	2	59.4 (10.5–108.3)	98.3%	<0.000
Home	4	56.8 (33.2–80.4)	99.4%	<0.000
Workplace	11	50.5 (37.2–63.9)	99.9%	<0.000

### Test for publication bias

**Figure 3:**
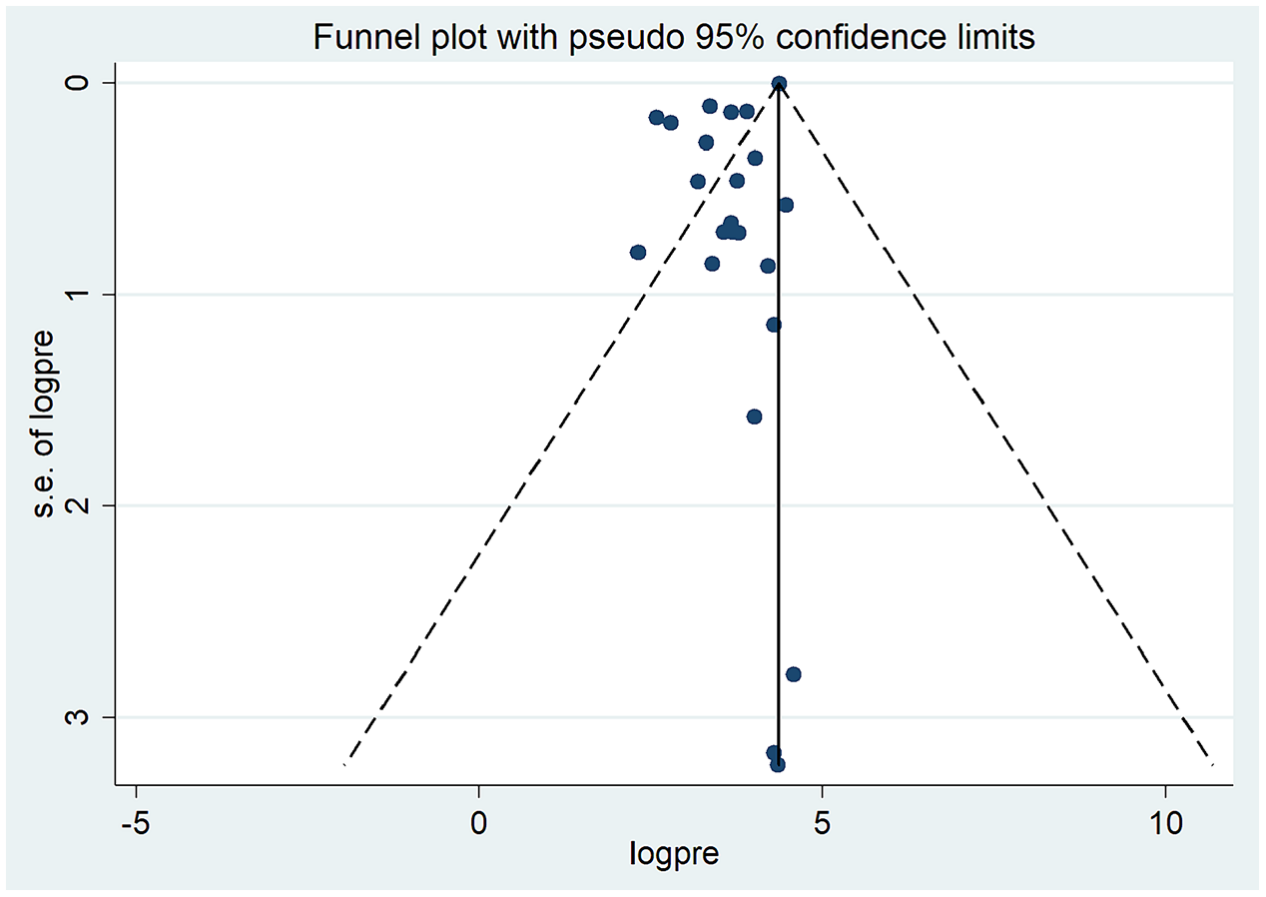
Funnel plot of the pooled smoke-free law non-compliance in public places, 2023.

**Figure 4:**
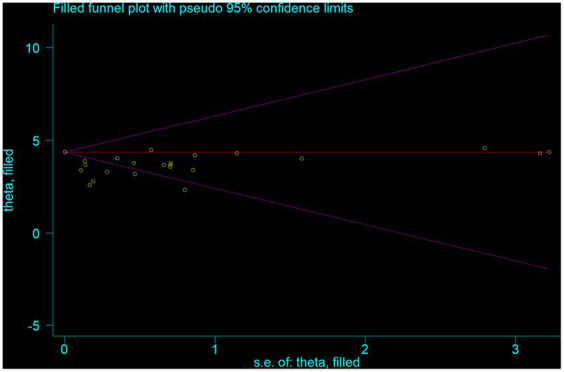
The funnel plot of a simulated meta-analysis.

Funnel plot revealed the presence of significant publication bias (Figure 3). The Egger test statistics also revealed the presence of statistically significant publication bias (*p* = 0.002). To determine the sources of this bias, a trim and fill analysis was conducted, revealing notable variation in the newly estimated pooled odds ratio, denoted as the adjusted point estimate [OR = 3.56, (95% CI: 3.19–3.94)], when compared to the initial or observed point estimate [OR = 4.36, (95% CI: 4.35–4.37)] (Figure 4).

### Subgroup analysis

**Figure 5:**
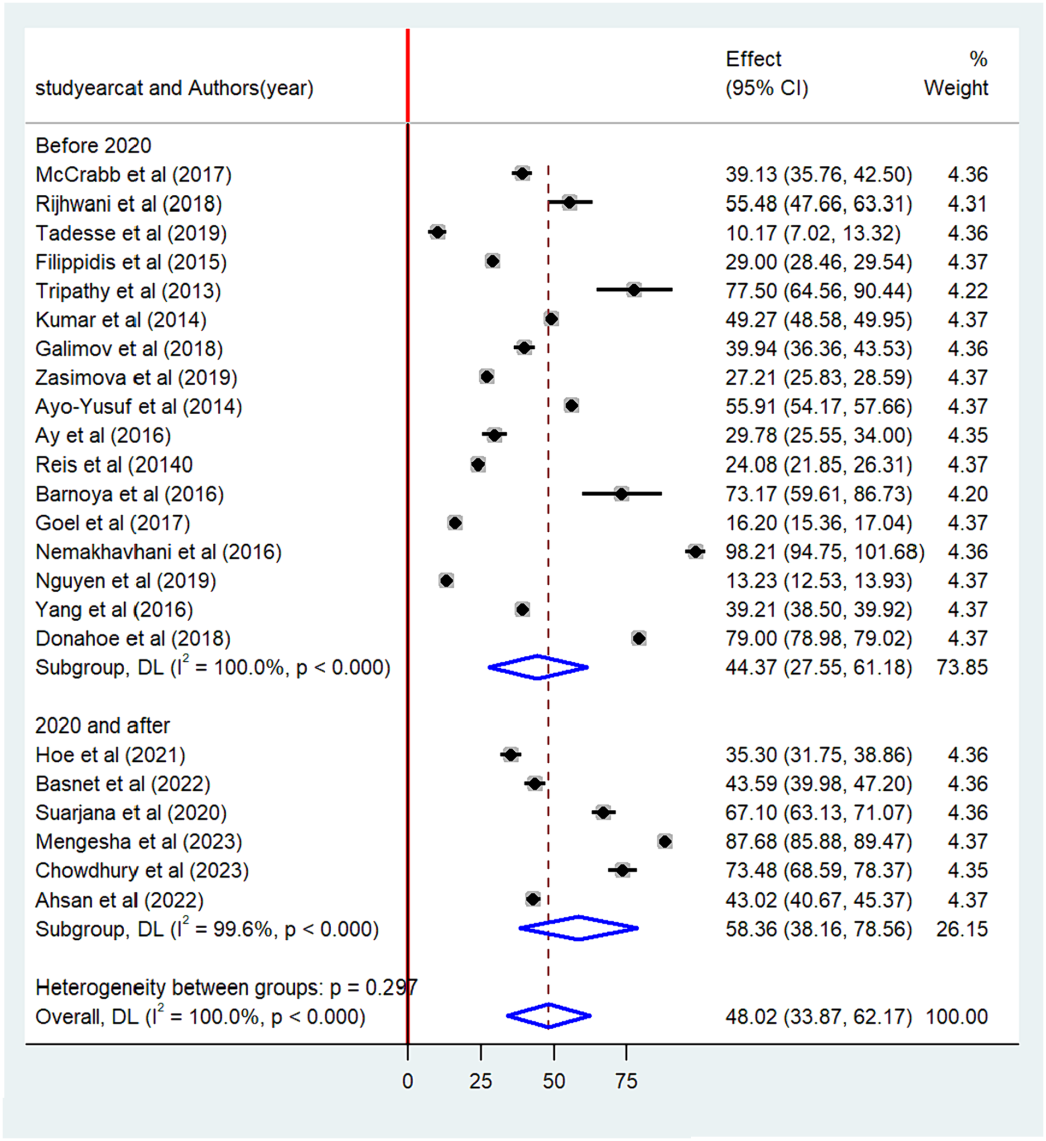
Subgroup analysis by the year of publication.

**Figure 6:**
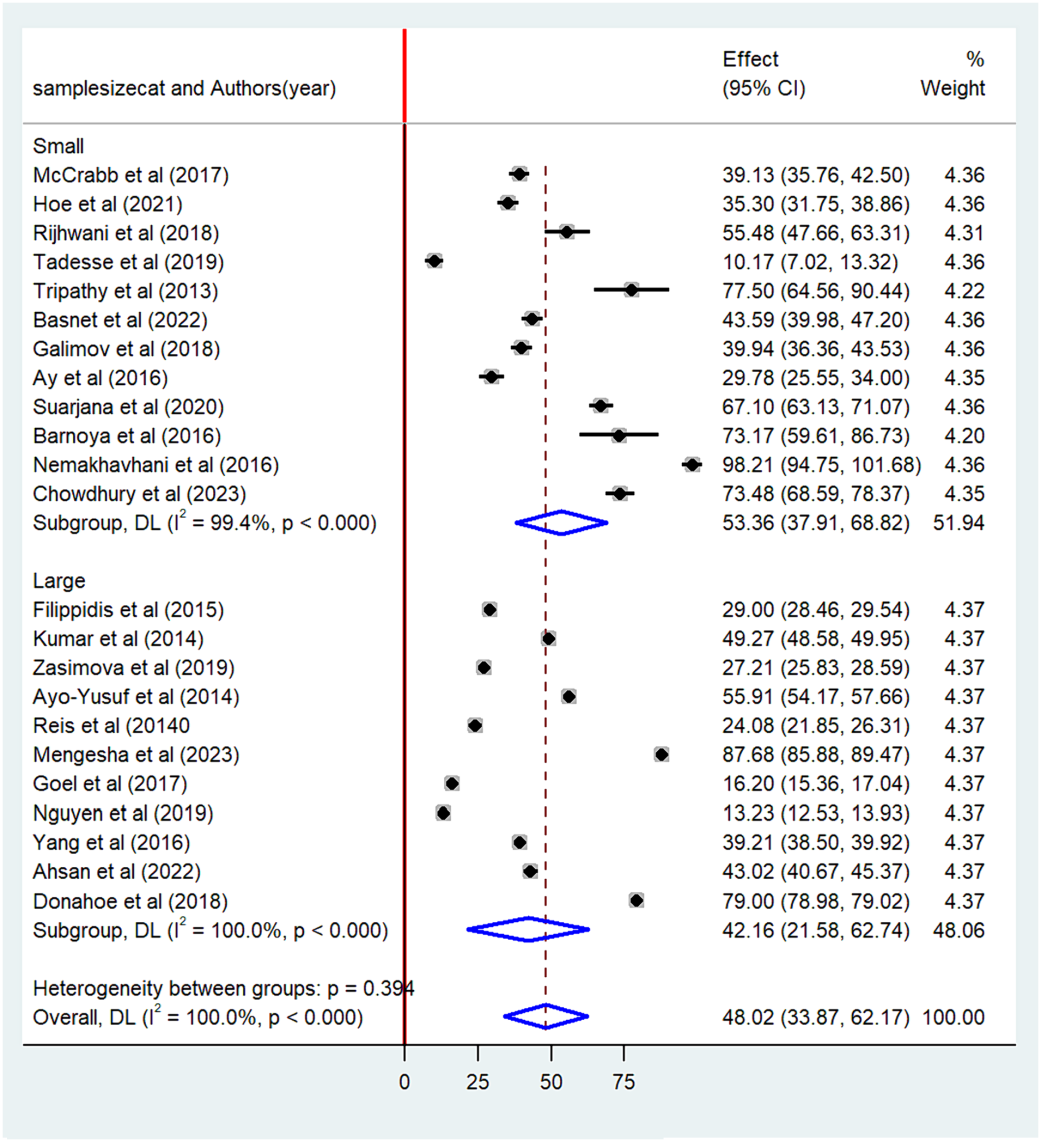
Subgroup analysis by sample size.

**Figure 7:**
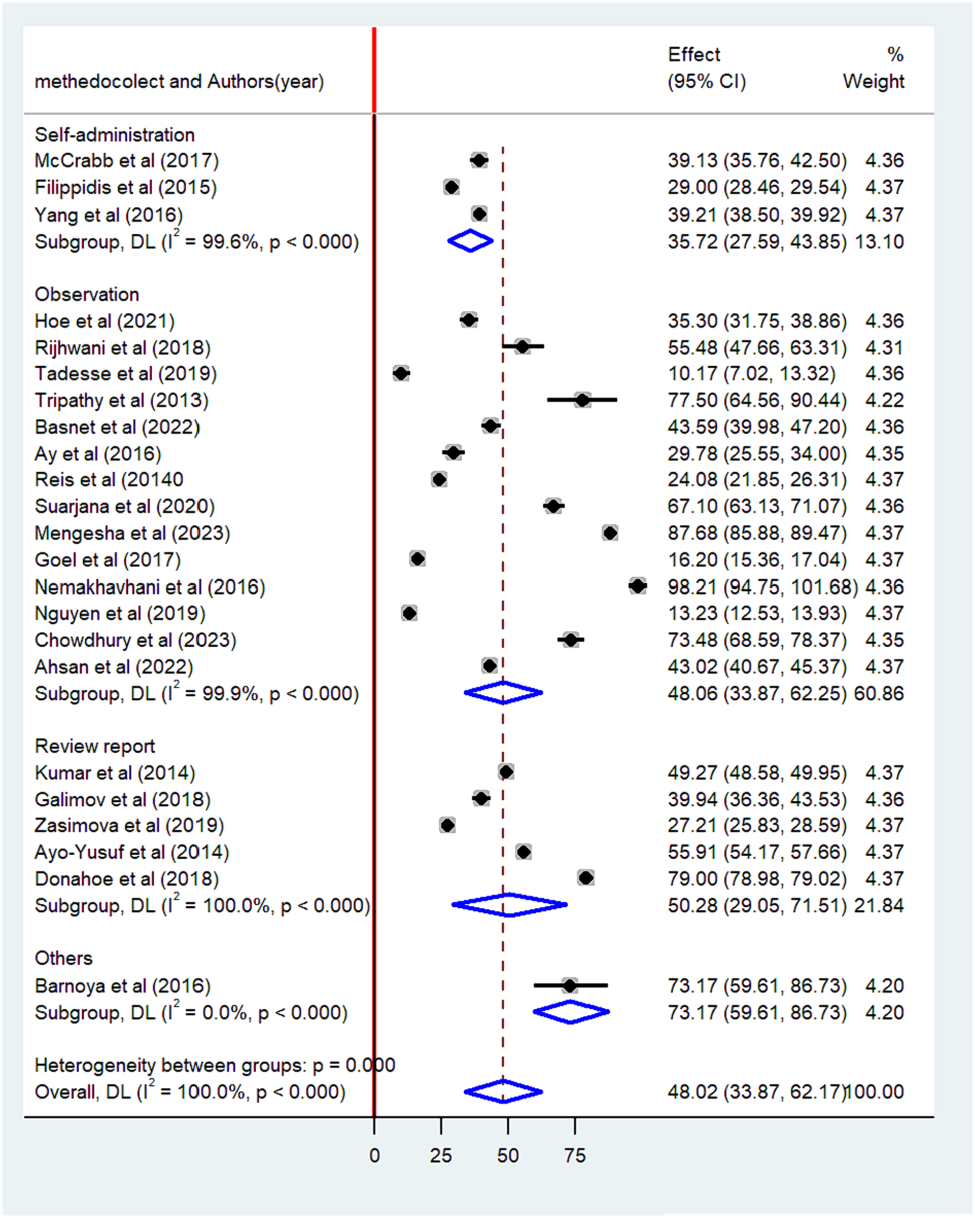
Subgroup analysis by the method of data collection.

The pooled prevalence of non-compliance with smoke-free law was found to be higher among studies conducted after 2020 (58.4, 95% CI: 36.16–78.56) than studies conducted before 2020 (44.4, 95% CI: 27.55–61.18) (Figure 5). When categorized based on the sample size, the highest prevalence of non-compliance with smoke-free law was reported among studies with a small sample size (53.4, 95% CI: 39.91–68.82) as compared to studies with a high sample size (42.2, 95% CI: 21.58–62.74) (Figure 6). Regarding the methods of data collection, the highest pooled non-compliance was observed among studies conducted by review report (50.3, 95% CI: 29.05–71.51) followed by observational studies (48.1, 95%CI: 33.87–62.25) (Figure 7).

### Meta-regression and sensitivity analysis

**Figure 8:**
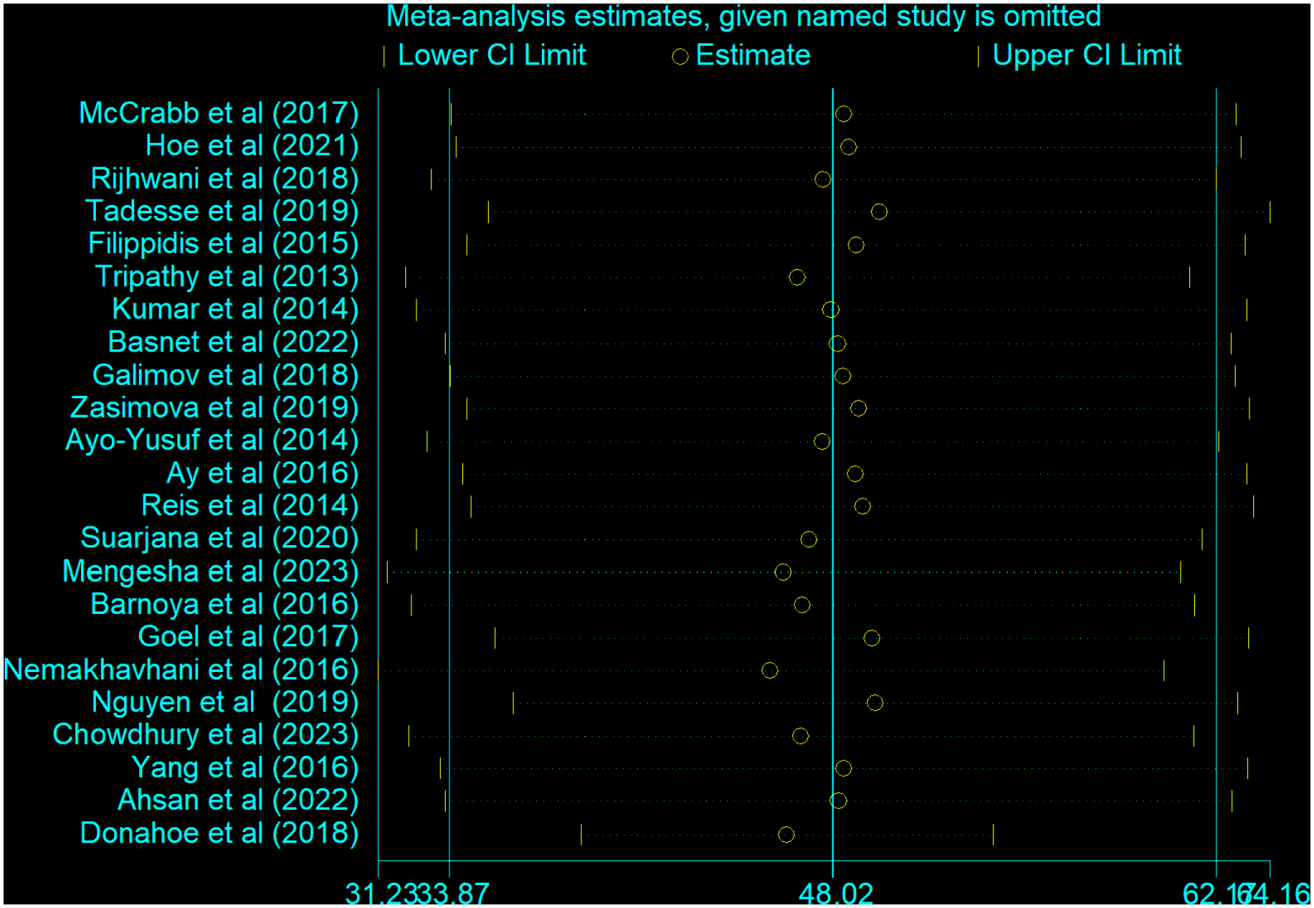
Sensitivity analysis of the included studies.

A univariate meta-regression model was carried out to pinpoint the source of heterogeneity by considering methods of data collection, year of publication, and sample size as factors. However, none of these variables demonstrated statistical significance (Table 3). Furthermore, a sensitivity analysis was performed to evaluate the impact of individual studies on the overall pooled estimate of non-compliance. The results also indicated no single study effect (Figure 8).

**Table 3:** Univariate meta-regression analysis to identify factors associated with the heterogeneity of the prevalence of non-compliance smoke-free law, 2023.

Variables	Coefficient	*p*-value
Year of publication	0.614517	0.394
Sample size	0.0456745	0.949
Methods of data collection	0.3194294	0.221

## Discussion

Exposure to secondhand smoke continues to be one of the leading causes of preventable mortality and morbidity, which might be due to the lack of smoke-free environment. The current meta-analysis showed that the pooled prevalence of non-compliance with the smoke-free law was 48.02% (95% CI: 33.87–62.17). This study indicates that almost half of the venues did not comply with the smoke-free law, which is considered as a violation of fundamental human rights and the tobacco smoke policy of the WHO framework convention (46, 47).

Evidence from the meta-analysis showed that hotels were the most frequently visited places that had high violations of smoke-free law (59.4%) followed by homes (56.8%) and workplaces (50.5%). The high non-compliance of smoke-free law in those public places might be attributed to the fact that governmental officials and policy-makers who are responsible for upholding policies remain reluctant to implement effective monitoring at the administrative level. In addition, failure to respect the law of the land and the absence of political will could be mentioned as the reasons for high non-compliance (48, 49). According to the surgeon general report in the US, exposure to secondhand smoke in the workplace is linked to an increased risk of non-communicable diseases among non-smoker workers in different public places (50).

On the other hand, education sector had the lowest non-compliance (35.2%) with smoke-free legislation, with statistically significant heterogeneity. Personnel in education sector might actively create awareness about adherence to smoke-free legislation among their students and the whole staff. Although this systematic review and meta-analysis was conducted according to the updated preferred reporting items for a systematic review and meta-analysis (PRISMA) guideline, it included only fourteen countries. Therefore, it might not be representative of the global data. All the articles included in this study were also cross-sectional studies, which could limit the causality of predictors.

## Conclusion

Non-compliance with smoke-free law in public places was found to be high. Public places, such as food and drinking establishments and healthcare facilities had the highest non-compliance, which calls for urgent intervention. This study indicated that it is not enough to pass smoke-free legislation; adequate implementation and enforcement of smoke-free legislation by public policymakers is essential to protect the health of adolescents, students and the whole community as well. Therefore, it is recommended that respective nations and stakeholders need to urgently adopt and enforce the comprehensive package of the WHO convention on tobacco control focusing on priority public places.

## Data availability statement

The original contributions presented in the study are included in the article/Supplementary material, further inquiries can be directed to the corresponding author.

## Author contributions

CD: Conceptualization, Data curation, Formal analysis, Funding acquisition, Investigation, Methodology, Project administration, Resources, Software, Supervision, Validation, Visualization, Writing – original draft, Writing – review & editing. AA: Formal analysis, Investigation, Methodology, Resources, Software, Supervision, Validation, Visualization, Writing – review & editing. KG: Data curation, Investigation, Methodology, Project administration, Resources, Validation, Visualization, Writing – review & editing. AG: Data curation, Formal analysis, Investigation, Methodology, Project administration, Visualization, Writing – review & editing. MG: Investigation, Methodology, Project administration, Resources, Software, Supervision, Validation, Visualization, Writing – review & editing.
