# Supplementary material for: Non-compliance with smoke-free law in public places: a systematic review and meta-analysis of global studies
Source: Front Public Health. 2024 Apr 17;12:1354980. doi: 10.3389/fpubh.2024.1354980 (PMC11061889; doi:10.3389/fpubh.2024.1354980)
Supplement: Supplementary file 1 [file Table_1.DOCX]

Hinari: ((non-compliance) OR (compliance)) AND ((smoke free law) OR (smoke free legislation) OR (smoking-ban) OR (smoking ban)) AND ((associated factors) OR (factors) OR (determinant factors) OR (factors associated)) AND ((public institution) OR (home) OR (restaurant) OR (cafe) OR (hospitals)) OR (schools)) OR (bar)) OR (bar and restaurant)) OR (public place))

Pubmed: (((((((((((((((((non-compliance) OR (compliance)) AND (smoke free law)) OR (smoke free legislation)) OR (smoking-ban)) OR (smoking ban)) AND (associated factors)) OR (factors associated)) OR (factors)) OR (determinant)) AND (public place)) OR (public institution)) OR (home)) OR (restaurant)) OR (bar and restaurant)) OR (hospitals)) OR (schools)) OR (bars) AND ((ffrft[Filter]) AND (2000/1/1:2023/11/16[pdat])) OR "Guideline Adherence"[MeSH Terms] AND "Tobacco Control"[MeSH Terms] AND "Risk Factors"[MeSH Terms]
