# Supplementary material for: Non-compliance with smoke-free law in public places: a systematic review and meta-analysis of global studies
Source: Front Public Health. 2024 Apr 17;12:1354980. doi: 10.3389/fpubh.2024.1354980 (PMC11061889; doi:10.3389/fpubh.2024.1354980)
Supplement: Supplementary file 3 [file Table_3.docx]

**Results of JBI Quality Assessment**

| Studies | Clear eligibility criteria | Description of study subject and study setting | Valid and reliable method to measure the exposure | Standard criteria used for measurement of the condition | Identification of confounding factors | Develop of strategies to deal with confounding factors | Valid and reliable method to measured outcomes | Appropriate statistical analysis | Total score out of 8 | Level of bias (%) |
| --- | --- | --- | --- | --- | --- | --- | --- | --- | --- | --- |
| McCrabb et al | Yes | Yes | No | No | Yes | Yes | Yes | Yes | 6 | 75 |
| Hoe et al | Yes | Yes | Yes | Yes | Yes | Yes | No | Yes | 7 | 87.5 |
| Rijhwani et al | Yes | Yes | Yes | No | Yes | Yes | Yes | Yes | 7 | 87.5 |
| Tadesse et al | Yes | Yes | Yes | Yes | Yes | Yes | Yes | Yes | 8 | 100 |
| Filippidis et al | Yes | Yes | yes | No | Yes | No | Yes | No | 5 | 62.5 |
| Tripathy et al | Yes | Yes | Yes | No | Yes | Yes | Yes | Yes | 7 | 87.5 |
| Kumar et al | Yes | Yes | Yes | Yes | Yes | No | Yes | Yes | 7 | 87.5 |
| Basnet et al | Yes | Yes | Yes | Yes | Yes | Yes | Yes | Yes | 8 | 100 |
| Galimov et al | Yes | Yes | No | Yes | Yes | Yes | Yes | Yes | 7 | 87.5 |
| Zasimova et al | Yes | Yes | Yes | Yes | Yes | No | Yes | Yes | 7 | 87.5 |
| Ayo-Yusuf et al | Yes | No | Yes | Yes | Yes | No | Yes | Yes | 6 | 75 |
| Ay et al | Yes | Yes | No | Yes | Yes | No | Yes | Yes | 6 | 75 |
| Reis et al | Yes | Yes | Yes | Yes | Yes | No | Yes | Yes | 7 | 87.5 |
| Suarjana et al | Yes | No | Yes | Yes | Yes | No | Yes | No | 5 | 62.5 |
| Mengesha et al | Yes | Yes | Yes | Yes | Yes | Yes | Yes | Yes | 8 | 100 |
| Donahoe et al | Yes | Yes | Yes | Yes | Yes | No | No | No | 5 | 62.5 |
| Barnoya et al | Yes | Yes | Yes | Yes | Yes | No | Yes | Yes | 7 | 87.5 |
| Goel et al | Yes | Yes | Yes | Yes | Yes | No | Yes | Yes | 7 | 87.5 |
| Nemakhavhani et al | Yes | Yes | Yes | No | Yes | No | Yes | Yes | 6 | 75 |
| Nguyen et al | Yes | Yes | Yes | No | Yes | Yes | No | Yes | 6 | 75 |
| Chowdhury et al | Yes | Yes | Yes | Yes | Yes | Yes | Yes | Yes | 8 | 100 |
| Yang et al | No | yes | yes | No | Yes | yes | yes | Yes | 6 | 75 |
| Ahsan et al | Yes | Yes | Yes | Yes | Yes | No | Yes | Yes | 7 | 87.5 |
